# Cancer screening and prevention in BRCA mutation carriers: a missed opportunity?

**DOI:** 10.1038/s41416-019-0484-8

**Published:** 2019-06-07

**Authors:** Nathalie LeVasseur, Stephen Chia

**Affiliations:** Department of Medical Oncology, British Columbia Cancer Agency, 600 West 10th Avenue, Vancouver, BC V5Z 4E6 Canada

## Abstract

While the elevated lifetime risk of breast and ovarian cancer is well recognised for patients with a BRCA mutation, the implementation of effective risk reduction strategies has been fraught with challenges. This report from an international database and published in the *British Journal of Cancer* reveals suboptimal rates of utilisation of surveillance/preventative measures globally.

## Main

While the majority of breast cancers are sporadic, it is estimated that ~5-10% of breast cancers are due to pathogenic mutations in BRCA1 and BRCA2.^1^ Given the high lifetime risk of developing breast and ovarian cancers in individuals with these carriers, the early adoption of preventative care and surveillance strategies is of utmost importance. Despite the availability of national and international guidelines to guide prevention efforts,^2,3^ physicians' attitudes towards preventative therapeutic interventions, such as prophylactic bilateral mastectomy, have been variable.^4–6^ Furthermore, high-risk screening and prevention efforts are challenging to implement in a younger target population, and are often overseen by primary care providers who may not be privy to the most recent and rapidly changing recommendations. Few reports of the uptake of these preventative measures longitudinally and across countries are available.

In this issue of the *British Journal of Cancer*, Metcalfe et al.^7^ report the international trends in the uptake of cancer risk-reduction strategies in women with a BRCA1 or BRCA2 mutation. Women were recruited from an international database of BRCA mutation carriers in 59 centres, across 10 countries, with data for a mean follow-up time of 7.5 years and a minimum of 1.5 years. A questionnaire was administered at the time of genetic testing and then twice yearly thereafter. Data sets from an earlier report published in 2008^5^ were included and compared with this expanded and contemporary cohort from 2009 onwards. Furthermore, analyses of geographic location and comparisons between time cohorts were carried out. A total of 6226 women met the pre-specified inclusion criteria. The median age of the study population was 52.1 years (27–96 years), including 42.3% with a prior diagnosis of a unilateral breast cancer. Women with a history of cancer other than unilateral breast cancer were excluded. Furthermore, women diagnosed with breast cancer during the follow-up period were also excluded. Outcomes of interest were prophylactic risk-reducing surgeries, including bilateral mastectomy and bilateral salpingo-oophorectomy (BSO), chemoprevention with tamoxifen or raloxifene, and breast cancer screening with mammography and/or MRI.

The study,^7^ run by the Hereditary Breast Cancer Clinical Study Group, offers insight into the uptake of preventative strategies globally for women with a BRCA mutation who carry a high lifetime risk of breast and ovarian cancer. One of the particular strengths of the study is the ability to compare the utilisation of preventative strategies longitudinally to further understand the impact of an evolving prevention/surveillance landscape for women with a BRCA mutation. With regard to risk-reducing surgeries, 27.9% underwent bilateral prophylactic mastectomy, with the highest rates in the USA (at 49.9%) and the lowest in Poland (at 4.5%). Furthermore, 62.8% went on to have prophylactic BSO, which was consistent over time, with a mean age of 45.6 years at the time of BSO. Chemoprevention remained a minimally utilised preventative strategy, with reported rates of 6.3%, although its use was notably higher in the USA (at 14.7%). Finally, with regard to breast screening, the uptake of mammography was 82.1% and decreased over time, whereas rates of MRI screening increased over time, up to 81.3% in the contemporary cohort.

This report^7^ is one of the few to compare and contrast global trends of preventative strategies within a high-risk group of women with deleterious BRCA mutations. While the Hereditary Breast Cancer Clinical Study Group should be acknowledged for their co-ordinated efforts in reporting the data relative to breast cancer, it is interesting that only patients with a prior history of breast cancer were enrolled in the study, since many BRCA mutations are detected at the time of diagnosis of epithelial ovarian cancer (EOC).^8,9^ Furthermore, although rates of breast cancer after a diagnosis of EOC are lower than the opposite sequence, the reported rates of uptake of breast cancer screening strategies in patients with EOC remain suboptimal, with a 2014 report suggesting rates of annual mammography of 59.3%, annual MRI of 44.4% and rates of prophylactic bilateral mastectomy of 9.6%.^10^

It is also interesting to note that women who were diagnosed with breast cancer at the time of enrolment into the study were excluded from future analyses, as the uptake of BSO for that population would have been an interesting metric to capture. In fact, in patients with a prior history of unilateral breast cancer included in this study,^7^ rates of BSO were 70.7% after a diagnosis of breast cancer, despite data showing that EOC can be reduced by more than 90% when performed before menopause.^11,12^ It remains unknown if greater knowledge of the updated guidelines would have translated into higher rates of uptake in this patient population. While the suboptimal uptake is undoubtedly reflective of patient preference, physicians should emphasise to patients that there are no effective screening strategies for EOC, which is associated with a higher stage at presentation and high morbidity and mortality.^13^ Moreover, 73.4% of patients included in this study had a BRCA1 mutation,^7^ which is associated with earlier onset of EOC compared with BRCA2,^9^ although the mean age of BSO for patients with a BRCA1 mutation was 44.7 years in this study.

Finally, this study^7^ highlighted a number of trends, which should be recognised. MRI screening for high-risk individuals is supported by most guidelines for patients with a BRCA mutation.^2,3^ However, while MRI has been effective in conjunction with mammography as a screening tool to reduce breast cancer mortality,^14,15^ evidence supporting a survival advantage with MRI screening alone is lacking. The authors^7^ demonstrated that the uptake of MRI has increased overtime, with a parallel reduction in mammography. However, guidelines suggest that a screening approach that includes annual MRI starting at 25 years of age, in combination with mammography starting at 30 years of age, is probably most effective at balancing the risks and benefits of screening.^2,3,16^ It is not clear from the details provided whether the lower rates of mammography are related to a younger age at the time of genetic testing or if there was an overall decrease in mammography across all cohorts. Even in the country with the lowest reported rates of mammography, mean age at the time of baseline interview was 46.6 years. Ultimately, the risk of breast cancer in those at 70 years of age with a BRCA mutation is in the order of 45–65% and the risk of contralateral breast cancer as high as 80%,^17,18^ which highlights the need for effective and standardised high-risk screening protocols that can be adopted outside a tertiary cancer care centre.

Research in the field of cancer genetics is evolving and preventative measures, including prophylactic surgery and high-risk screening protocols, are constantly being refined to integrate novel findings that can be applied to clinical practice. It is therefore important that studies like these are conducted to evaluate the real-world effectiveness of preventative efforts and also to avoid missed opportunities.

## Data Availability

Not applicable
